# London Practice

**Published:** 1872-10

**Authors:** James Perrigo

**Affiliations:** England, Demonstrator of Anatomy, University of Bishop's College


### SELECTIONS.

---

### LONDON PRACTICE.

BY JAMES PERRIGO, A.M., M.D., M.R.C.S., ENGLAND,
*Demonstrator of Anatomy, University of Bishop's College.*

At the Samaritan Female Hospital I had the pleasure of seeing Mr. Spencer Wells operate for ovariotomy nine times. He advises the operation to be performed as early as possible, as then the tumor is less vascular, and consequently there is less likelihood of there being adhesions. He invariably brings the pedicle to the surface, and secures it there by the clamp. Mr. Wells has now operated four hundred times, and as he finished each hundred, he brought the results before the Medico-Chirurgical Society.

In the first hundred, thirty-four died; in the second, twenty-eight died; in the third, twenty-three died; in the fourth, twenty-two died.

Of the last hundred, forty-four were in the hospital, and fifty-six in private practice. He has hopes of reducing the mortality to ten per cent. He adds that the mortality in hospital and private practice is about the same. In his opinion, tappings do not considerably increase the mortality, and sometimes are of benefit in giving time for the health to improve, or in lessening the shock by having fluid removed a few days before. I may state that when the pedicle is of sufficient length, the clamp is always preferred. Mr. Gant, who is senior surgeon to the Royal Free Hospital, thinks the probability of success is nearly doubled, where the length of pedicle permits this arrangement.

At the West London Hospital I saw Dr. Wiltshire amputate the vaginal part of the cervix uteri in two cases. The cervix in each case was enormously elongated, so much so, as to protrude to the vulva. They both did well. The instrument employed was the écrasuer, but instead of being armed with the chain-saw, it had a single wire. It is much more easily used, makes a cleaner cut, and prevents bleeding as effectually.

At Moorfield's Ophthalmic Hospital, a person can have every facility in studying eye cases. Every surgeon has, on an average, one hundred to one hundred and fifty out-door patients to prescribe for, and there are four of them busy doing so, every morning from nine to noon, after which there is always some operation to be performed. A stranger there is treated with all possible kindness, and every attention is shown to him in explaining in the opthalmoscopic room. This room is quite a large one, darkened and fitted up with nine stalls, each of which is furnished with a gas burner, two chairs and a small table. Some days, during the out-door hours, there is hardly one of these unoccupied for a moment. There are many Canadians who, no doubt, will remember with gratitude the great kindness there exhibited toward them; even sometimes firm and lasting friendships are formed. It was so in my case, and in after life, I shall always look back upon my visit to London with pleasure. I was fortunate enough to be allowed to examamine a case of opaque optic nerve fibres, a condition that is not to be seen every day. Tonics, rest and fomentations, formed the general run of treatment. Rheumatic ophthalmia is more common in England than here. It is treated chiefly with aconite and colchicum, along with the bi-carbonate of potash. Mercury is given largely in syphilitic iritis, just as long as any lymph is formed. Von Gräefe's operation for cataract is the favorite method. About seventy-five per cent. of the cases do well.

In visiting any of the London hospitals, one is astonished with the neatness and regularity with which everything is done. All the hospitals have assistant physicians and surgeons to attend to the out-door work. The posts of house-physicians and house-surgeons have to be competed for by examination, and they are not held longer than six months. All the nurses are trained by the Sisters of St. John, and the hospitals that take nurses from this institution pay it so much a year. Charing Cross pays £500. In addition, there is a Sister to each ward, with a nurse under her, and over the whole hospital is a Sister who looks after the diet and wine cellar.

A great deal of treatment in the London hospitals is highly experimental. Calabar bean was tried for chorea at the Children's Hospital, Great Ormond Street. As might be expected, it produced no effect on the disease, but, strange to relate, it dilated the pupil. Bromide of potassium is now given for profuse menorrhagia, instead of other remedies. Since my return I have tried it successfully in one case. The galvano-cautery is now pretty generally employed, and I have seen it used for those little vascular tumors at the meatus-urinarius of the female. Carbolic acid, as a dressing, is extensively used in some of the hospitals, but the cases are better chosen for its application than I have seen elsewhere. Holmes Coote will not allow any of it in his wards, and there are a few others who will not use it. Craniotomy is now being gradually supplanted by cephalotripsy. In displacements of the uterus, Grailey Hewitt's ring pessaries are almost universally used, as they can be bent to any desired shape. Lallemand's porte-caustique is now improved by Sir Henry Thompson and Erichsen. Their instruments are so made that caustics in a fluid condition can be applied. There is very little difference between Sir Henry's and Erichsen's. Each consists of a catheter, the lower portion being perforated by minute holes. In it is a stilette, having at the bottom a sponge fitted on a spring attached to the stilette; the sponge is pressed upon, and the fluid is squeezed out.

Some of the professional men in London, after their appointment to a hospital, however small the institution, consider it their duty to write a book for the edification of their less fortunate brethren. The result of this is a vast amount of medical and surgical literature that falls still-born from the press.

The amount of money given to charitable institutions in Great Britain is almost incredible. Much of it is mis-applied. There is a multiplicity of servants at the hospitals. It takes two or three porters to do work that could be performed here by one, and other items are nearly in proportion. In some institutions, the Board meets once every quarter, and sits down to a sumptuous dinner, the expenses being defrayed out of the Infirmary's income. At one of these board "feeds," of which I was myself cognizant, the wine alone cost £27. There are many other things of like character that remain unknown to the public. This was also the case with the Red Cross Society; a society that did much good, and yet was much cheated. Some of the London instrument-makers were enabled to sell a lot of old stock, useless for any practical purposes, to agents of the Society who had not the slighest idea what instruments were required. Comparing the Profession in Great Britain to that in Canada, I think we have nothing to be ashamed of. We have everything to encourage us, when we consider the differences of advantages; how wealthy their institutions are, and how poorly ours are endowed. They have one advantage over us, their men are generally better educated before they enter upon their professional studies, and in proportion to the number of schools, there are fewer testing bodies.

They are also better supporters of professional periodicals, and so far as I could judge, there seems to be a greater unanimity and esprit-de-corps, in trying to raise the standard of the Profession. The various "pathies" are let alone. Homœopathy, for instance, is never mentioned, and is not noticed at all.

Gratuitous advice at the different hospitals has been abused to such an extent that steps have been taken to have it remedied. Dr. Meadows and others relate cases where persons have dressed themselves in their servants' clothing, and have presented themselves for advice at the out-door departments. Well-to-do farmers come to London for the same purpose. I know of some farmers on the Island here, who do the same thing. Recently a society has been formed, called the Charity Organization. The committee rooms are in St. George's, Hanover Square. All suspected cases are referred to them for investigation. The University College Hospital has already referred a good many suspicious patients to the committee, and many glaring cases of imposition have been exposed. When it was discovered that these patients could well afford to pay for medical advice, they were referred to the general practitioners in the neighborhood of their abode. Some such arrangement might with propriety be instituted in Montreal.—*Canada Medical Journal, and Louisville and Richmond Medical Journal.*

